# Role of endothelial cells in pulmonary fibrosis via SREBP2 activation

**DOI:** 10.1172/jci.insight.125635

**Published:** 2021-11-22

**Authors:** Marcy Martin, Jiao Zhang, Yifei Miao, Ming He, Jian Kang, Hsi-Yuan Huang, Chih-Hung Chou, Tse-Shun Huang, Hsiao-Chin Hong, Shu-Han Su, Simon S. Wong, Rebecca L. Harper, Lingli Wang, Rakesh Bhattacharjee, Hsien-Da Huang, Zhen Bouman Chen, Atul Malhotra, Marlene Rabinovitch, James S. Hagood, John Y-J. Shyy

**Affiliations:** 1 Division of Cardiology, Department of Medicine, UCSD, La Jolla, California, USA.; 2 Vera Moulton Wall Center for Pulmonary Vascular Diseases,; 3 Stanford Cardiovascular Institute, and; 4 Department of Pediatrics, Stanford University School of Medicine, Stanford, California, USA.; 5 School of Life and Health Sciences, The Chinese University of Hong Kong, Shenzhen, Longgang District, Shenzhen, Guangdong Province, China.; 6 Warshel Institute for Computational Biology, and School of Science and Engineering, The Chinese University of Hong Kong, Shenzhen, Guangdong Province, China.; 7 Institute of Bioinformatics and Systems Biology, Department of Biological Science and Technology, National Chiao Tung University, Hsinchu, Taiwan.; 8 Department of Bioengineering and Institute of Engineering in Medicine and; 9 Division of Respiratory Medicine, Department of Pediatrics, UCSD, La Jolla, California, USA.; 10 Department of Diabetes Complications and Metabolism, Beckman Research Institute, City of Hope, Duarte, California, USA.; 11 Division of Pulmonary and Critical Care Medicine, UCSD, La Jolla, California, USA.; 12 Division of Pulmonology, Department of Pediatrics, University of North Carolina at Chapel Hill, Chapel Hill, North Carolina, USA.

**Keywords:** Pulmonology, Vascular Biology, Endothelial cells, Fibrosis

## Abstract

Idiopathic pulmonary fibrosis (IPF) is a progressive lung disease with limited treatment options. Despite endothelial cells (ECs) comprising 30% of the lung cellular composition, the role of EC dysfunction in pulmonary fibrosis (PF) remains unclear. We hypothesize that sterol regulatory element-binding protein 2 (SREBP2) plays a critical role in the pathogenesis of PF via EC phenotypic modifications. Transcriptome data demonstrate that SREBP2 overexpression in ECs led to the induction of the TGF, Wnt, and cytoskeleton remodeling gene ontology pathways and the increased expression of mesenchymal genes, such as snail family transcriptional repressor 1 (snai1), **α**-smooth muscle actin, vimentin, and neural cadherin. Furthermore, SREBP2 directly bound to the promoter regions and transactivated these mesenchymal genes. This transcriptomic change was associated with an epigenetic and phenotypic switch in ECs, leading to increased proliferation, stress fiber formation, and ECM deposition. Mice with endothelial-specific transgenic overexpression of SREBP2 (EC-SREBP2[N]-Tg mice) that were administered bleomycin to induce PF demonstrated exacerbated vascular remodeling and increased mesenchymal transition in the lung. SREBP2 was also found to be markedly increased in lung specimens from patients with IPF. These results suggest that SREBP2, induced by lung injury, can exacerbate PF in rodent models and in human patients with IPF.

## Introduction

Pulmonary fibrosis (PF) is characterized by areas of active myofibroblasts depositing extracellular matrix, which result in dysfunctional remodeling of lung parenchyma (1, 2). Lung injury has been hypothesized to cause an acute inflammatory response, followed by dysregulated wound repair with ensuing fibrosis in many PF disease entities, including idiopathic pulmonary fibrosis (IPF) (3). IPF is a devastating condition characterized by progressive deterioration in lung function in those afflicted. Newer IPF therapies, such as nintedanib and pirfenidone (4), slow disease progression but do not affect mortality; the only treatment option for many patients is lung transplantation (5). Attempts at reducing inflammation, blocking endothelin, or inhibiting phosphodiesterase type 5 (PDE5) have been unsuccessful, leading to ongoing efforts to identify new approaches to treatment (6, 7).

In lung specimens from patients with IPF, an increased number of myofibroblasts is observed within fibroblastic foci, in the interstitial space between the epithelium and the capillaries. The prototypical proliferative signals in these myofibroblasts include TGF-β and PDGF pathways, which also mediate exuberant secretion of collagen and fibronectin (FN) (8). Recent studies suggest these myofibroblasts can be derived not only from resident lung fibroblasts, but also from circulating fibrocytes or mesenchymal cells (9). Epithelial-to-mesenchymal transition (EMT) and endothelial-to-mesenchymal transition (EndoMT) have both been hypothesized to play a pathogenic role in the progression of fibrosis in many organs, including the lung (10–12). There is controversy in the literature regarding the concept of EndoMT (13, 14). In this study, we use the term partial EndoMT, recognizing that some cells have a mesenchymal phenotype while maintaining endothelial cell (EC) markers — for example, in myofibroblast-like cells. Regarding ECs, the capillary endothelium comprises one-third of the cells in the normal lung (15), but relatively little is known about the role of vascular ECs and partial EndoMT in the pathogenesis of lung injury and the development of PF.

Bleomycin (BLM) is a chemotherapy agent with a major side effect of inducing PF in approximately 10%–30% of patients (16–18). BLM reacts with oxygen to create superoxide, free radicals, and reactive oxygen species (ROS). Lung tissue lacks the enzyme that degrades BLM (19), causing its accumulation and resulting in pulmonary toxicity. Several mouse models of PF involve administration of BLM, either via the airway or systemically, to create lung injury and subsequent fibrosis. Stress-inducing stimuli such as TGF-β and ROS are known to promote lung injury and a mesenchymal phenotype, although the mechanisms remain poorly understood. Of note, we have previously found that the oxidative stress–responsive sterol regulatory element-binding protein 2 (SREBP2) mediates EC dysfunction in vascular diseases such as atherosclerosis (20, 21). SREBP2 is a transcription factor with its canonical function in regulating cholesterol biosynthesis and uptake. Although these functions have been well characterized, ECs do not readily uptake, nor do they accumulate cholesterol (22), which suggests that SREBP2 may be involved in additional mechanisms in EC biology.

We postulated that SREBP2 has a critical role in the pathogenesis of lung injury and fibrosis via EC phenotypic modifications, and we tested this hypothesis in rodent models and human tissue. Data collected from the current study demonstrate that SREBP2 was activated by BLM in ECs and promoted partial EndoMT in vitro and in vivo. SREBP2, functioning as a transcription factor, could transactivate mesenchymal genes such as snail family transcriptional repressor 1 (snai1), α-smooth muscle actin (αSMA), neural cadherin (N-Cad), and vimentin, which was mediated in part through epigenetic modulations. At the disease level, EC-specific SREBP2 overexpression in mice exacerbated BLM-induced PF, and this was consistent with the significantly elevated level of SREBP2 in lung specimens from patients with IPF.

## Results

### SREBP2 transactivates mesenchymal genes.

**Figure 1:**
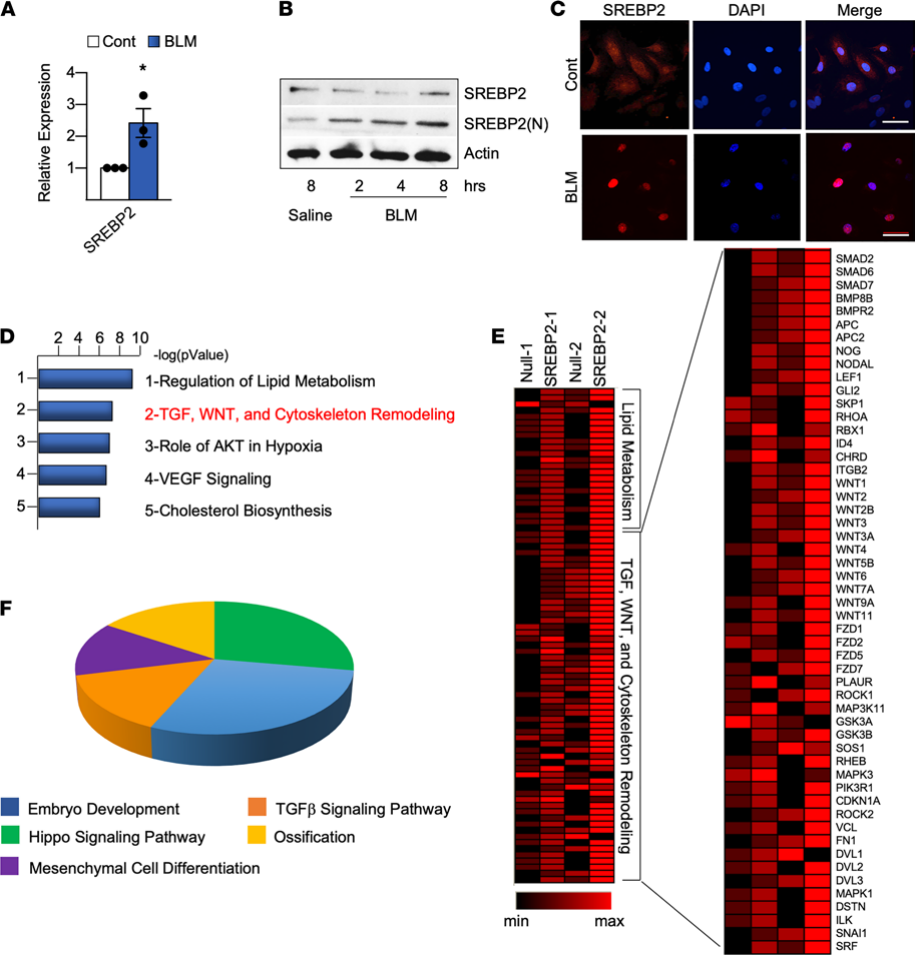
Bleomycin-induced (BLM-induced) SREBP2(N) mediates profibrotic pathways in ECs. (**A**–**C**) HUVECs were stimulated with BLM (1 mU). (**A**) Following 72 hours of BLM treatment, mRNA was measured using qPCR. (**B**) N-terminal cleavage of SREBP2 was measured using Western blot. (**C**) Representative image of nuclear localization was determined with immunofluorescence using anti-SREBP2 (red) and DAPI (blue) (*n* = 3). Scale bar: 50 μm. (**D**–**F**) HUVECs were infected with Ad-SREBP2(N) or Ad-null (empty vector) for 72 hours (*n* = 2). Total RNA was isolated and analyzed by RNA-Seq. (**D**) Gene ontology (GO) analysis was performed for the top 500 upregulated genes in ECs overexpressing SREBP2(N). (**E**) Heatmap indicates the upregulated genes of *n* = 2 data sets in the TGF, WNT, Cytoskeleton Remodeling pathways. (**F**) PANTHER analysis of the genes listed in **E**. Data in **A** were analyzed by 2-tailed Student’s *t* test, and data are represented as mean ± SEM from 3 independent experiments (*n* = 3). *P < 0.05 between the indicated groups. WNT, wingless related integration site.

Because SREBP2 plays a key role in oxidative stress–induced endothelial dysfunction (21), we first identified whether BLM could activate SREBP2 in ECs. As postulated, BLM increased SREBP2 expression, revealed by increased SREBP2 mRNA, and it increased SREBP2 activation, demonstrated by increased N-terminal cleavage (SREBP2[N]) and nuclear translocation in ECs (Figure 1, A–C, and Supplemental Figure 1; supplemental material available online with this article; https://doi.org/10.1172/jci.insight.125635DS1). Consistently, BLM increased redox burden in the endothelium (Supplemental Figure 2A). Given that BLM induced the transcription factor SREBP2 in ECs, we then profiled the transcriptome in ECs overexpressing SREBP2(N). Pathway enrichment analysis of the RNA-Seq data revealed that, as expected, lipid metabolism was the highest-ranked pathway (Figure 1D). The next highest–ranked pathway was that of TGF, Wnt, and cytoskeleton remodeling (Figure 1D). Transcripts in these pathways included snai1, mothers against decapentaplegic proteins (SMADs), bone morphogenetic protein (BMPs), and wingless-type mouse mammary tumor virus (MMTV) integration site family members (Wnts) (Figure 1E). Noticeably, these upregulated genes are mediators of embryonic development, mesenchymal cell differentiation, and Hippo signaling (Figure 1F), all of which are implicated in mesenchymal transition (23–25). Collectively, RNA-Seq and in silico analyses revealed that SREBP2(N) overexpression in ECs not only induced lipid metabolism, but also mediated a mesenchymal transcriptional program with activation of profibrotic pathways.

**Figure 2:**
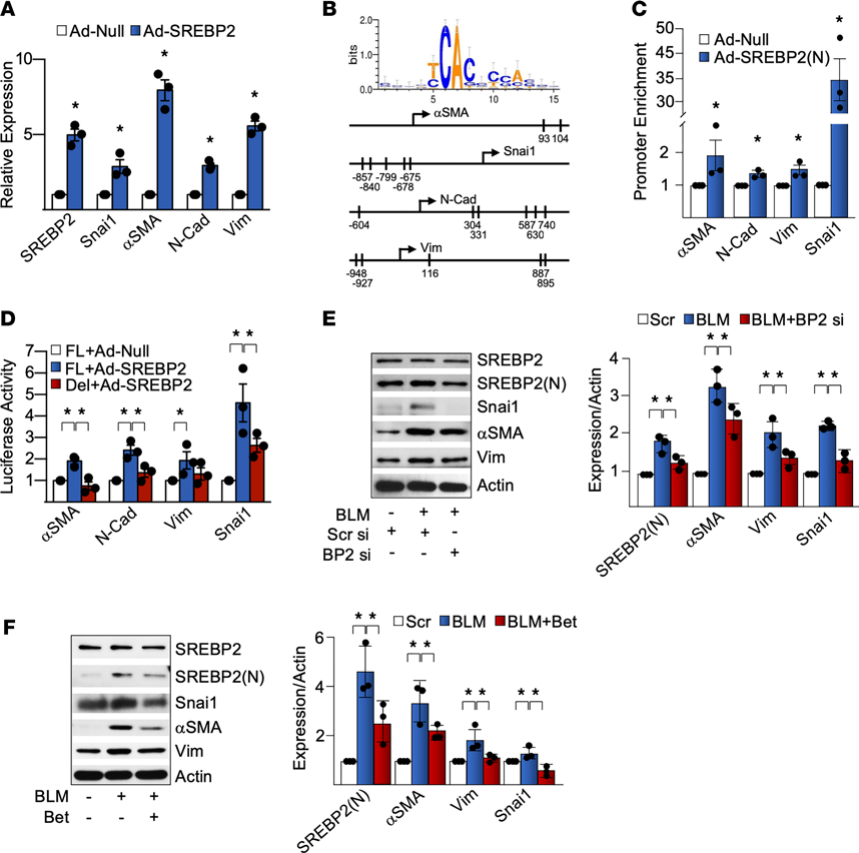
SREBP2 transactivates mesenchymal genes. (**A**) HUVECs were infected with Ad-SREBP2(N) or empty vector (Ad-null). The levels of mRNA of the indicated genes were measured by qPCR. (**B**) Depiction of the predicted sterol regulatory elements (SREs) in the promoter regions of snail family transcriptional repressor 1 (snai1), α-smooth muscle actin (αSMA), N-Cadherin (N-Cad), and vimentin (Vim). (**C** and **D**) HUVECs were infected with Ad-SREBP2(N) or empty vector (Ad-Null). (**C**) Chromatin immunoprecipitation (ChIP) assays were performed on the promoter region of *SNAI1* (encoding snai1), *ACTA2* (encoding αSMA), *CDH2* (encoding N-Cad), and *VIM* (encoding Vim). (**D**) Luciferase activity was measured and normalized to that of Renilla. FL, full length promoter; Del, SRE site deletion. (**E**) HUVECs were transfected with SREBP2 or scrambled control siRNA (20 nM) for 16 hours prior to stimulation with bleomycin (BLM) for an additional 48 hours. (**F**) HUVECs were pretreated with betulin (Bet) (6 μg/mL) for 2 hours, followed by BLM treatment for an additional 72 hours. The expression of the indicated proteins was measured by Western blot. Data in **A** and **C** were analyzed by 2-tailed Student’s *t* test; data are represented as mean ± SEM from 3 independent experiments (*n* = 3). Data in **D**–**F** were analyzed by 1-way ANOVA with Bonferroni post hoc; data are represented as mean ± SEM from 3 independent experiments (*n* = 3). *P < 0.05 between the indicated groups.

Validating these results with quantitative PCR (qPCR), we found that exogenous expression of SREBP2(N) indeed augmented the mRNA levels of snai1, αSMA, N-Cad, and vimentin (Figure 2A and Supplemental Figure 2B) in ECs. We speculated that SREBP2 may directly transactivate mesenchymal genes in ECs. In agreement with this hypothesis, the promoter regions of *SNAI1*, *ACTA2* (αSMA), *CDH2* (N-Cad), and *VIM* (vimentin) genes were predicted by using MATCH (26) to contain conserved SREBP2 binding sites — i.e., sterol responsive elements (SREs) (Figure 2B). ChIP assays revealed that ECs overexpressing SREBP2(N) had increased SREBP2 binding to the promoter region of the respective mesenchymal markers (Figure 2C). Furthermore, using respective promoter regions fused to the luciferase reporter, we confirmed that SREBP2 transactivated the promoter regions of *SNAI1*, *ACTA2*, *CDH2*, and *VIM* genes, which was inhibited when the putative SREs were deleted (Figure 2D). Because SREBP2(N) was sufficient to potentiate mesenchymal transition, we then investigated whether BLM could induce mesenchymal genes and if this was dependent on SREBP2. BLM treatment indeed increased the expression of mesenchymal genes (Supplemental Figure 2C). In ECs in which SREBP2 was knocked down by siRNA or pharmacologically inhibited by betulin (a pan-SREBP inhibitor), BLM induction of snai1, αSMA, and vimentin was attenuated (Figure 2, E and F). Taken together, Figure 2 suggests that BLM-induced SREBP2 was both necessary and sufficient to transactivate mesenchymal markers in ECs.

### SREBP2 modulates chromatin accessibility.

**Figure 3:**
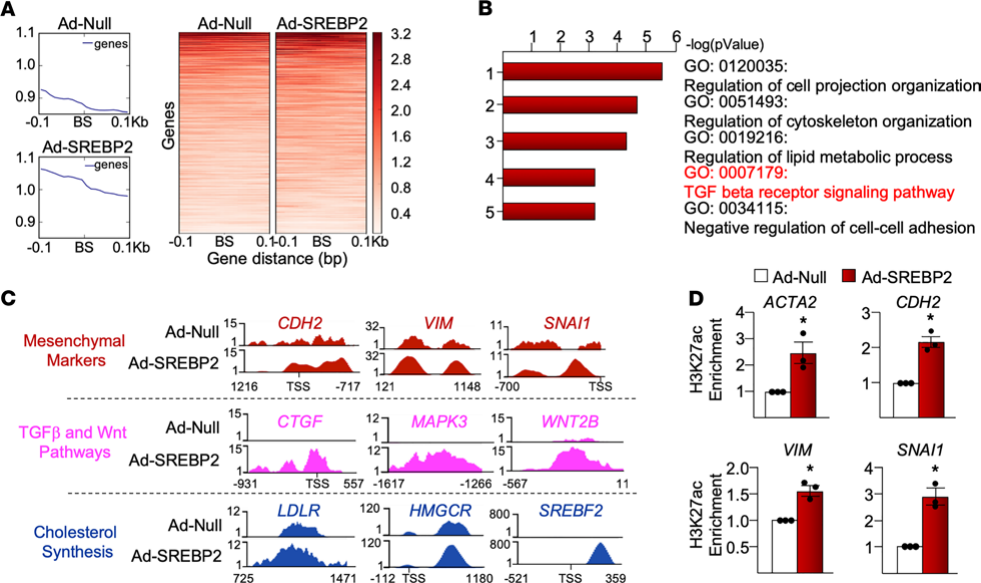
ATAC-Seq reveals SREBP2 modulation of the chromatin accessibility. HUVECs were infected with Ad-SREBP2(N) or empty vector (Ad-Null) followed by ATAC-Seq (*n* = 2). (**A**) ATAC peaks were merged into a union set. SREBP2 putative binding sites within the ATAC peaks were predicted. The upper and lower panels show overall and per-loci ATAC signals at ± 0.1 kb flanking the putative SREBP2 binding sites (BS), respectively. (**B**) Genes specific to mesenchymal markers and TGF-β/Wnt pathways indicate chromatin decondensation upon SREBP2(N) overexpression by using WashU Epigenome Browser. Cholesterol biosynthesis genes were used as a positive control. (**C**) Gene ontology (GO) analysis reveals top activated pathways with SREBP2(N) transactivation. (**D**) H3K27ac ChIP-qPCR indicates the chromatin remodeling of the mesenchymal genes. Data in **D** were analyzed by 2-tailed Student’s *t* test; data are represented as mean ± SEM from 3 independent experiments (*n* = 3). *P < 0.05 between the indicated groups. ACTA2, α smooth muscle actin; CDH2, neural cadherin; CTGF, connective tissue growth factor; HMGCR, 3-Hydroxy-3-Methylglutaryl-CoA Reductase; LDLR, low density lipoprotein receptor; MAPK3, mitogen activated protein kinase 3; SNAI1, snail family transcriptional repressor 1; TGF, transforming growth factor; VIM, vimentin; Wnt, wingless integration site.

Because cell transdifferentiation is regulated in part by epigenetic modulations involving chromatin remodeling (27), we then performed an assay for transposase-accessible chromatin sequencing (ATAC-Seq) to assess chromatin accessibility in ECs overexpressing SREBP2(N). Condensed chromatin regions exhibit attenuated ATAC-Seq peaks and are less accessible to transcriptional machinery, whereas decondensed chromatin shows large peaks and indicates increased chromatin accessibility (28). As shown in Figure 3A, loci containing putative SREs at the genome-wide scale were largely decondensed in ECs overexpressing SREBP2(N) when compared with control ECs. This suggests that SREBP2 increased chromatin accessibility of its target genes. Pathway enrichment analysis revealed that loci with significant changes in ATAC-Seq signals were associated with genes involved in pathways such as TGF-β receptor signaling pathways and lipid metabolic process (Figure 3B). With respect to the upstream region of individual genes, SREBP2(N) overexpression increased chromatin accessibility of the mesenchymal genes (e.g., *SNAI1*, *VIM*, *CDH2*), those involved in TGF-β/Wnt pathways (e.g., *CTGF*, *MAPK3*, *WNT2B*), and cholesterol biosynthesis (e.g., *LDLR*, *HMGCR*, *SREBP2*) (Figure 3C). Active and open chromatin states were further assessed by using Histone 3 Lys 27 acetylation (H3K27ac) ChIP-qPCR. As shown in Figure 3D, the upstream region of *SNAI1*, *ACTA2*, *VIM*, and *CDH2* exhibited H3K27ac enrichment in ECs overexpressing SREBP2(N). These data suggest that SREBP2 binding to the promoter regions of *SNAI1*, *ACTA2*, *CDH2*, and *VIM* led to increased chromatin accessibility.

### SREBP2 suppresses endothelial markers.

**Figure 4:**
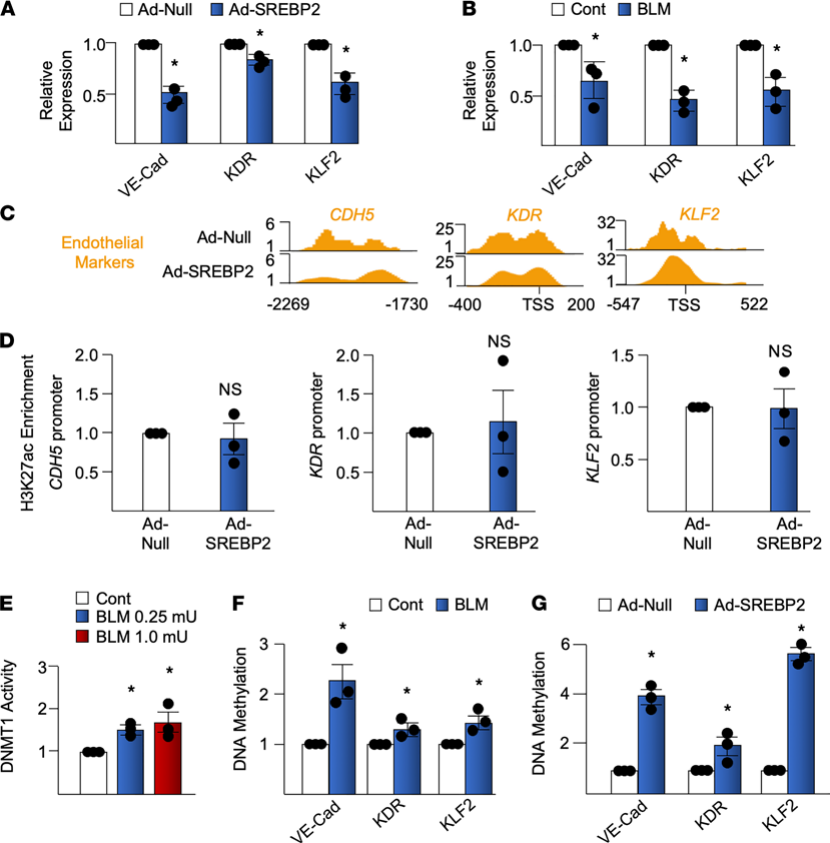
BLM-induced SREBP2 suppresses EC markers. (**A**) HUVECs were treated with BLM (1 mU) for 72 hours. (**B**) HUVECs were infected with Ad-SREBP2(N) or empty vector (Ad-Null) for 72 hours. The mRNA expression levels of VE-Cadherin (VE-Cad), kinase insert domain receptor (KDR), and Krüppel-like factor 2 (KLF2) were measured using qPCR. (**C**) ATAC-Seq indicates the chromatin state of genes specific to EC markers upon SREBP2(N) overexpression. (**D**) H3K27ac ChIP-qPCR showing the chromatin state of indicated genes following 72 hours of Ad-SREBP2(N) infection compared with empty vector. (**E**) HUVECs were treated with the indicated concentration of BLM. DNA methyltransferase 1 (DNMT1) activity was measured using ELISA. (**F** and **G**) HUVECs were treated with BLM or infected with Ad-SREBP2(N) or Ad-Null for 72 hours. Isolated DNA was bisulfite converted and subjected to methylation-specific qPCR. Data in **A**, **B**, and **D**–**G** were analyzed by 2-tailed Student’s *t* test; data are represented as mean ± SEM from 3 independent experiments (*n* = 3). *P < 0.05 between the indicated groups.

EndoMT is characterized not only by the gain of mesenchymal markers, but also by the reciprocal loss of EC markers. As postulated, SREBP2(N) overexpression suppressed the expression of EC markers (i.e., Krüppel-like factor 2 [KLF2], vascular endothelial cadherin [VE-Cad], and vascular endothelial growth factor receptor 2 [VEGFR2] also known as kinase insert domain–containing receptor [KDR] in humans) (Figure 4A). In parallel experiments, BLM treatment also suppressed these EC markers (Figure 4B). This indicates that the EC-to-mesenchymal genotypic switch was induced by BLM and SREBP2, hallmarked by the increased mesenchymal markers and reduced EC markers. With decreased expression of EC markers at the mRNA level, the promoter regions of these genes exhibited marginal change in chromatin accessibility, as indicated by ATAC-Seq and H3K27ac ChIP-qPCR (Figure 4, C and D). These data suggest that chromatin accessibility might not be directly involved in SREBP2 suppression of EC markers. We therefore further analyzed the promoter region of the ECs markers by exploring DNA methylation, which is in part regulated by DNA methyltransferase 1 (DNMT1) (29). In agreement with previous findings that DNMT1 expression is increased in patients with PF (30, 31), BLM treatment increased DNMT1 activity in ECs (Figure 4E). Furthermore, using methylation-specific (MSP) qPCR, we found that BLM treatment, consistent to that of SREBP2(N) overexpression, caused DNA hypermethylation of EC promoters (Figure 4, F and G). Together, results in Figure 4 demonstrate the role of SREBP2 in genotypic modulation of EC markers, in part due to epigenetic modulations, such as DNA hypermethylation.

### SREBP2 promotes a myofibroblast-like phenotypic switch in vitro.

**Figure 5:**
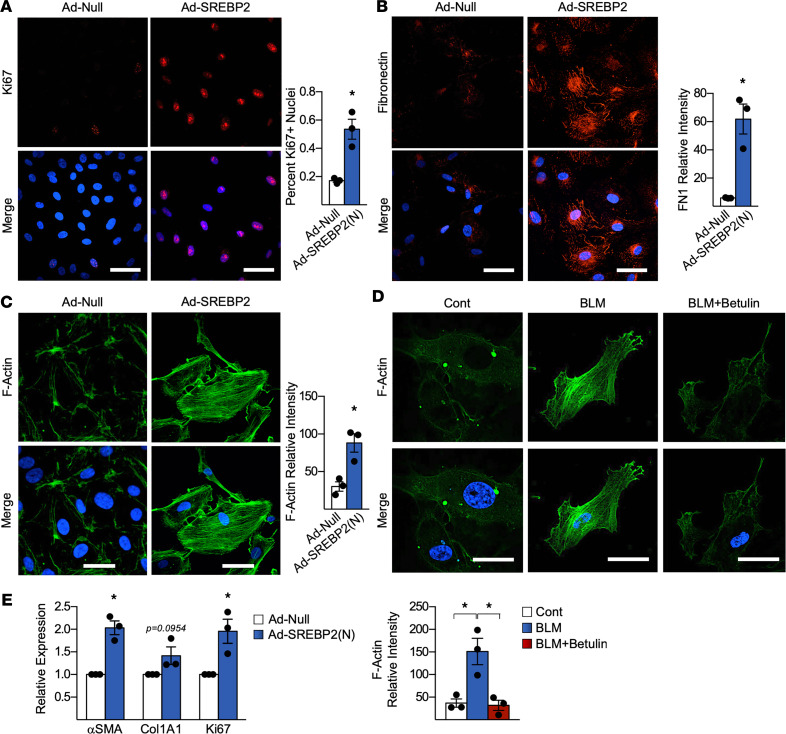
SREBP2 promotes a phenotypic switch in ECs that activated fibroblasts via paracrine effects. (**A**–**C**) Human umbilical vein ECs (HUVECs) infected with Ad-SREBP2(N) or empty vector (Ad-null) were analyzed by immunostaining. Proliferation was assessed using anti-Ki67 (red) (**A**), ECM deposition was observed using anti-fibronectin (red) (**B**), and stress fiber formation was examined using F-actin (green) (**C**). In all experiments, nuclei were counter stained with DAPI (blue). (**D**) HUVECs were pretreated with betulin for 2 hours, followed by bleomycin (BLM) for 72 hours. Stress fiber formation was assessed using F-actin (green). Nuclei were counter stained with DAPI (blue). Scale bar: 50 μm. (**E**) Human lung microvascular ECs (HLMECs) were infected with Ad-SREBP2(N) or Ad-Null, and they were then cocultured using a transwell system with human lung fibroblasts for 72 hours. mRNA expression was measured using qPCR. Data in **A**–**E** were analyzed by 2-tailed Student’s *t* test; data are represented as mean ± SEM from 3 independent experiments (*n* = 3). *P < 0.05 between the indicated groups. Col1A1, collagen 1 type 1; FN1, fibronectin 1.

We next examined whether the aforementioned transcriptional and epigenetic alterations corresponded to changes in endothelial biological function and, hence, phenotypic alterations. To study the phenotypic switch in ECs resulting from the activation of the BLM/SREBP2/EndoMT axis, we examined proliferation, ECM deposition, and stress fiber formation in ECs overexpressing SREBP2(N) (32, 33). As shown in Figure 5A, SREBP2(N) overexpression increased proliferation by 40%, revealed by the Ki67 immunostaining. Furthermore, SREBP2(N) overexpression in ECs resulted in increased FN deposition and stress fiber formation (microfilament bundles as indicated by F-actin), consistent with a myofibroblast-like phenotype (Figure 5, B and C) (34). Stress fiber formation was also seen in BLM-treated ECs (Figure 5D). However, betulin cotreatment abolished actin fiber reorganization (Figure 5D). Additionally, SREBP2(N) was overexpressed in human lung microvascular ECs and then cocultured with human lung fibroblasts to assess the paracrine effect from ECs. Induction of SREBP2(N) in the microvascular ECs led to activation of the cocultured myofibroblast, indicated by an increase in αSMA and collagen expression, as well as a more proliferative phenotype (Figure 5E). In summary, BLM induction of SREBP2 in cultured ECs was associated with phenotypic changes, including increased proliferation, ECM deposition, and stress fiber formation. Furthermore, this change in EC phenotype rendered cocultured fibroblasts to become myofibroblasts via paracrine effects.

### SREBP2 promotes partial EndoMT contributing to PF in mouse.

**Figure 6:**
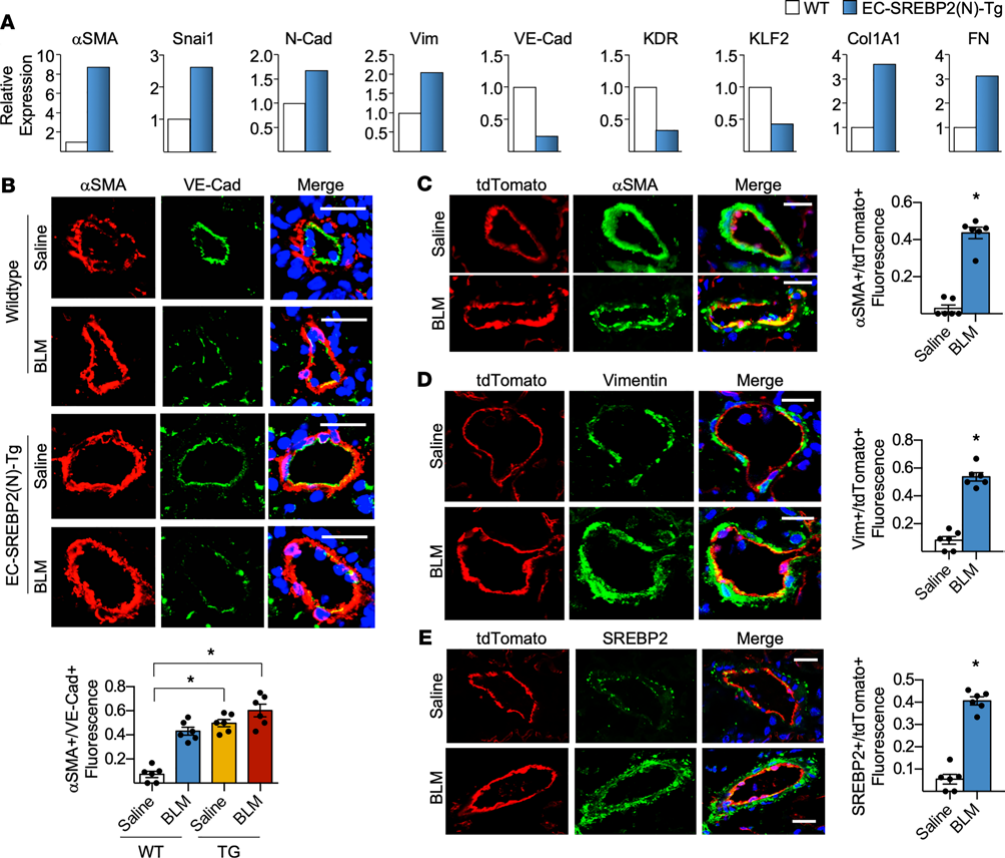
BLM-induced SREBP2 promotes EC phenotypic switch and partial EndoMT in mice. (**A**) Lung microvascular ECs were isolated from age-matched EC-SREBP2(N)-Tg mice and compared with their WT littermates (pooled from *n* = 5 per group). The level of indicated mRNA was measured using qPCR. (**B**–**E**) Age-matched EC-SREBP2(N)-Tg and WT littermates, or EC-specific tdTomato-expressing mice driven with tamoxifen inducible VE-Cad–Cre (EC-tdTomato mice) were administered BLM (8 units/injection) on days 0, 4, 7, 10, and 14 through i.p. injection. Twenty-eight days after BLM administration, lungs were harvested. (**B**) Frozen lung sections were immunostained for the EC marker VE-Cad (green) and the mesenchymal marker αSMA (red). Nuclei were counter stained with DAPI (blue). Scale bar: 20 μm. Quantitative analysis showing the number of αSMA^+^ cells compared with total VE-Cad^+^ cells in the intima is graphed below the representative images. (**C**–**E**) Lineage-tracing experiments were performed with EC-specific expression of tdTomato (red), counterstained with αSMA (green) (**C**), vimentin (green) (**D**), or SREBP2 (green) (**E**). Nuclei are labeled with DAPI (blue). Scale bar: 20 μm. Quantitative analysis showing αSMA^+^, Vim^+^, or SREBP2^+^ cells are compared with total tdTomato^+^ cells in the intima, which is graphed on the right of the representative images. Data in **B** were analyzed by 2-way ANOVA with Kruskal-Wallis post hoc; data are represented as mean ± SEM from *n* = 6 mice per group. Data in **C**–**E** were analyzed by 2-tailed Student’s *t* test; data are represented as mean ± SEM from *n* = 6 mice per group. *P < 0.05 between the indicated groups. Col1A1, collagen 1 type 1; FN1, fibronectin 1; KDR, kinase insert domain receptor; KLF2, Krüppel-like factor 2; N-Cad, neural cadherin; Snai1, snail family transcriptional repressor 1; VE-Cad, vascular endothelial cadherin; Vim, vimentin; Wnt, wingless integration site).

To identify the role of SREBP2-induced EndoMT at the tissue level in vivo, we utilized a mouse line that overexpresses SREBP2(N) in ECs (EC-SREBP2[N]-Tg mice) (20). Lung ECs isolated from EC-SREBP2(N)-Tg mice demonstrated increased mesenchymal markers (i.e., αSMA, snai1, N-Cad, and vimentin) and suppressed EC markers (i.e., eNOS, VE-Cad, and von Willebrand factor [vWF]), together with increased ECM genes (i.e., Col1A1 and FN), when compared with ECs isolated from age-matched WT mice (Figure 6A). This result indicates that SREBP2(N) overexpression, even in the absence of any stimulus, was sufficient to promote partial EndoMT at the tissue level. Finding that SREBP2 promoted an endothelial-to-mesenchymal–like transcriptional program in vivo, we next assessed whether BLM-induced PF is exacerbated in EC-SREBP2(N)-Tg mice. Following BLM or saline administration, lungs were collected for immunohistological analyses. Consistent with Figure 6A, WT mice treated with saline exhibited the highest expression level of VE-Cad (green) but the lowest expression level of αSMA (red), evidenced by the merged image having largely green staining in the pulmonary vessels (Figure 6B). Interestingly, WT mice treated with BLM and EC-SREBP2(N)-Tg mice treated with saline had a similar reduction in VE-Cad staining (Figure 6B). Notably, the BLM-treated EC-SREBP2(N)-Tg mice had a significant suppression of VE-Cad but the greatest increase in αSMA (Figure 6B), which was demonstrated by the orange staining in the merged image. To further delineate the role of ECs in the progression of PF in vivo, we performed an EC lineage-tracing study by creating a VE-Cad–specific tdTomato reporter mouse (EC-tdTomato mice). The tdTomato fluorescence was compared with that of VE-Cad in the mouse lung tissue to validate the specificity of this EC reporter model (Supplemental Figure 3). We then treated these reporter mice with or without BLM for 28 days and identified mesenchymal transition via the colocalization with αSMA, vimentin, and SREBP2 using immunostaining (Figure 6, C–E). The tdTomato signal was not found outside of the vessel wall with concurrent mesenchymal marker expression, suggesting a partial EndoMT phenotype.

**Figure 7:**
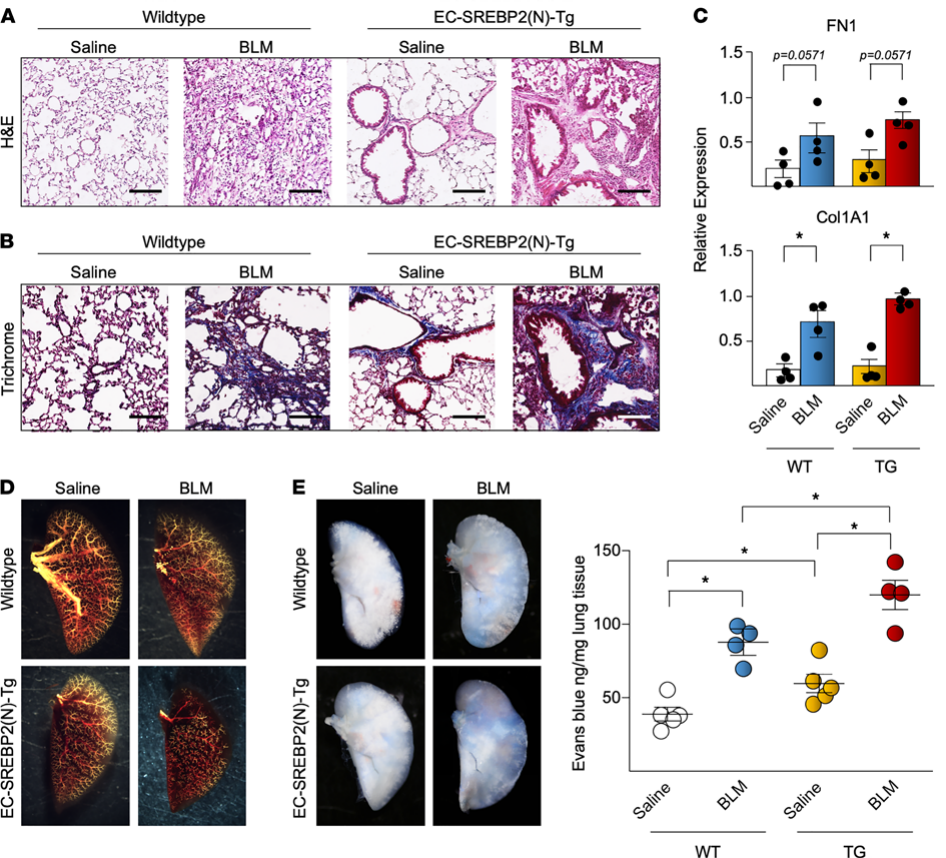
SREBP2 promotes EC-induced vascular damage in the mouse lung. (**A** and **B**) Vascular remodeling resulting from bleomycin (BLM) administration and SREBP2(N) overexpression was revealed by representative images of H&E staining from *n* = 6 mice per group (cells stained in red, nuclei in black) (**A**) and representative images of Masson’s trichrome from *n* = 6 mice per group (cells stained in red, collagen in blue) (**B**). Scale bars: 100 μm. (**C**) The level of fibronectin (FN1) and collagen (Col1A1) mRNA from whole lung samples were measured using qPCR. (**D**) Representative images of an angiogram with the use of Microfil to reveal the lung vascularization (yellow) (*n* = 4 mice per group). (**E**) Evans blue was injected via tail vein, and mice were then sacrificed 30 minutes later (*n* = 4 mice per group). Evans blue effusion in the lung is visualized in the representative images and were quantified by spectrophotometry. Data in **C** were analyzed by 2-tailed Student’s *t* test; data are represented as mean ± SEM from *n* = 4 mice per group. Data in **E** were analyzed by 2-way ANOVA with Kruskal-Wallis post hoc; data are represented as mean ± SEM from *n* = 4 mice per group. *P < 0.05 between the indicated groups.

We also examined the morphological changes associated with the BLM/SREBP2/EndoMT axis in the context of PF. H&E staining demonstrated a marked increase in mononuclear cell infiltration in the EC-SREBP2(N)-Tg mice treated with BLM (Figure 7A). Consistently, Masson’s trichrome staining demonstrated the increased collagen deposition in areas surrounding lung vasculature, as well as parenchymal fibrotic tissue (Figures 7B). qPCR assays of whole lung tissue further confirmed the increased collagen and FN content in the EC-SREBP2(N)-Tg mice treated with BLM when compared with WT mice receiving saline, WT mice receiving BLM, and EC-SREBP2(N)-Tg mice receiving saline (Figure 7C). To depict the vascularization in the lung, angiography illustrated that WT mice treated with BLM and EC-SREBP2(N)-Tg mice treated with saline had reduced distal vascularization, whereas the EC-SREBP2(N)-Tg mice treated with BLM had a severe reduction in small to medium vessels (Figure 7D). We also performed Evans blue dye staining to identify EC damage and permeability resulting from SREBP2 induction in the fibrotic lung. As shown in Figure 7E, BLM administration increased vascular leakage in mice, while the SREBP2(N)-Tg mice had the most severe effusion. Overall, data in Figure 6 and Figure 7 demonstrate that activation of SREBP2(N)-mediated partial EndoMT in mice worsened the BLM-induced PF, in part through a dysfunctional endothelium.

### SREBP2 and partial EndoMT are augmented in IPF lung.

**Figure 8:**
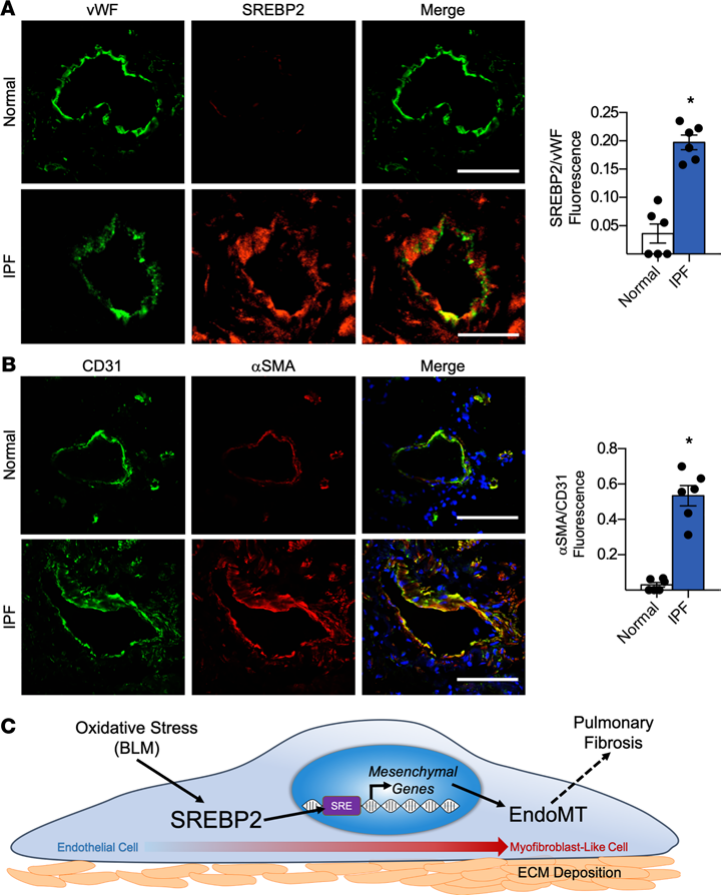
SREBP2 and mesenchymal markers are induced in human lung endothelium with idiopathic pulmonary fibrosis (IPF). (**A** and **B**) Human lung tissue samples from patients with IPF were compared with normal lung transplant controls (*n* = 6 per group). Immunostaining was performed with the endothelial cell marker vWF (green) and SREBP2 (red) (**A**), or the endothelial cell marker CD31 (green) and mesenchymal marker αSMA (red) (**B**). In all images, nuclei were counter stained with DAPI (blue). Scale bar: 20 μm. Data in **A** and **B** were analyzed by 2-tailed Student’s *t* test; data are represented as mean ± SEM from *n* = 6 patient samples per group. *P < 0.05 between the indicated groups. BLM, bleomycin; CD31, PECAM-1; EndoMT, endothelial-to-mesenchymal transition; vWF, von Willebrand factor. (**C**) The involvement of BLM/SREBP2/EndoMT axis in the lung ECs during the onset of PF.

To examine whether the deduced BLM/SREBP2/EndoMT axis is evident in human IPF, we explored pulmonary vessels isolated from patients with IPF. Consistent with our rodent PF model, pulmonary vasculature from patients with IPF had increased SREBP2 expression, which was localized to the nuclei, when compared with specimens from normal controls (Figure 8A). In line with augmented SREBP2, IPF subjects had increased partial EndoMT, as revealed by the increased αSMA colocalization with the EC marker CD31 (Figure 8B). Collectively, data in Figure 8 provide convincing evidence that SREBP2 may play a role in partial EndoMT contributing to PF in humans.

## Discussion

Despite the fact that ECs account for more than one-third of all normal lung cells (15), the role of impaired vascular endothelium in PF remains largely unknown. Our novel findings demonstrate that SREBP2 is integral to transcriptomic, epigenetic, and phenotypic changes in EC in response to profibrotic stresses in vitro and in vivo — findings that have translational implications in the pathogenesis of IPF. High-throughput screening, including RNA-Seq and ATAC-Seq, provided a global view of SREBP2 activation in ECs, which was concomitant with mesenchymal transition. Further in silico and in vitro validations revealed that SREBP2 directly transactivated the promoter regions of *SNAI1*, *ACTA2*, *CDH2*, and *VIM*. This genotypic switch led to a phenotypic change with increased proliferation, FN deposition, and stress fiber formation; thus, it yielded a myofibroblast-like cell phenotype. In vivo, BLM-induced PF was exacerbated in EC-SREBP2(N)-Tg mice at least in part via increased EC damage and subsequent myofibroblast-like cell transition. The translational implications of these findings build upon SREBP2 activation promoting a mesenchymal shift seen in specimens from patients with IPF. A summary of these findings is illustrated in Figure 8C.

With its canonical role in governing cholesterol biosynthesis, SREBP2 modulates cellular cholesterol homeostasis in many cell types, including hepatocytes and adipocytes (35, 36). We have previously shown that various forms of oxidative stress activate SREBP2 in the endothelium, and SREBP2 in turn induces the NLR family, pyrin domain containing protein 3 (NLRP3) inflammasome (20, 21). IPF has an unknown etiology but is thought to have an initial inflammatory and redox response followed by chronic fibrosis (1, 37). Intratracheal administration of BLM is the most commonly used method to induce PF in mice (38). However, this method does not resemble the onset of IPF in humans, as the initial damage is more inflammatory and bronchiocentric (39). A systemic administration method, such as i.p. injection used in the current study, exhibits primarily subpleural collagen deposition, which is more similar to the pathology seen in patients with IPF (38). Given that positive SREBP2 staining was observed in lung specimens from human IPF (Figure 7A) and that BLM-induced PF was worsened in EC-SREBP2(N)-Tg mice (Figure 6B), the findings strongly suggest that SREBP2 plays a central role in PF-related lung damage. The effects of inflammatory cytokines — such as IL-1β, the product of the inflammasome — have been previously shown to induce EndoMT in an NF-κB–dependent manner (40, 41), and we have previously shown that SREBP2 also induces IL-1β via inflammasome induction (20). Thus, SREBP2 may synergistically regulate EndoMT through direct transactivation of mesenchymal genes and indirectly through induction of inflammatory cytokines.

Exogenous overexpression of SREBP2 was sufficient to transactivate mesenchymal targets (e.g., snai1, αSMA, N-Cad, and vimentin) in vitro. Furthermore, loss-of-function experiments in which SREBP2 was genetically knocked down or pharmacologically inhibited in cultured ECs abolished BLM-induced partial EndoMT. Although the expression of mesenchymal markers was elevated in EC-SREBP2(N)-Tg mice, we suggest that SREBP2 induces partial EndoMT contributing to PF in vivo. Thus, our results demonstrate that SREBP2 is both sufficient and necessary to induce EC-to-myofibroblast–like cells.

Although our data show that SREBP2 transactivated genes encoding mesenchymal markers and suppressed those for EC markers, immunostaining and lineage-tracing experiments in Figure 6 imply that ECs did not fully transform into mesenchymal cells. These findings suggest that ECs likely do not fully account for the myofibroblast population in the interstitial space of the lung in BLM-induced PF. However, ECs likely undergo partial EndoMT during the onset of SREBP2-induced PF. Although not migrating into the interstitial space, these myofibroblast-like ECs still affect cell proliferation and ECM protein expression in neighboring cells, including the resident fibroblast population, via paracrine signals (Figure 5E). Furthermore, we have demonstrated human lung microvascular ECs had increased mesenchymal marker expression in vitro (Figure 5E); however, evidence was lacking for EndoMT in the microvascular ECs in the lineage-tracing experiments in vivo. Interestingly, the majority of the fibrosis seen in vivo occurred in the bronchovascular bundles (Figure 7, A and B), suggesting that SREBP2-induced partial EndoMT of the larger vessels may contribute more to the fibrosis development in these regions. Similar effects have been demonstrated in EMT contributing to kidney fibrosis, during which the transdifferentiated mesenchymal cell maintains association with the basement membrane (42, 43). Although not contributing to the myofibroblast population, such partial EMT is critical in the pathogenesis of renal fibrosis. In regard to PF, SREBP2-dependent partial EndoMT may cause a profibrotic milieu to promote fibrosis in the lung. Recently, single-cell RNA-Seq was utilized to identify different cell subpopulations in human IPF (44). Although these authors did not fully describe the molecular differences of every cell type within the human IPF lung, their work indicates a clear distinction in donor versus IPF ECs (44). This provides further evidence toward our hypothesis that dysfunctional ECs contribute to IPF. This technology will provide future exploration to study the role of endothelial dysfunction in human IPF at the single cell level.

We have recently identified that SREBP2 is sufficient for the activation of NLRP3 inflammasome in ECs (20). Given that the major products of the NLRP3 inflammasome are IL-1β and IL-18, SREBP2 would be a potential mediator of both the innate immune response and oxidative stress in ECs. Likewise, BLM administration increased IL-1β and IL-18 expression in the serum and lungs of C57BL/6 mice (45). Furthermore, BLM-induced lung injury was significantly attenuated in mice with ablated caspase-1 or IL-18 (45), suggesting the importance of the SREBP2-mediated innate immune response in PF.

High levels of circulating cholesterol result in increased lung innate immune response via immune cell infiltration, as well as activation of the NLRP3 inflammasome through TLR signaling (46, 47). The link between dysregulated cholesterol in cardiovascular and liver diseases has been studied intensively. However, the relationship between dyslipidemia and lung disease has only recently emerged (48). Furthermore, mice that lack enzymes needed for reverse cholesterol transport, such as ATP-binding cassette A1 (ABCA1) and ABCG1, both of which are downregulated by SREBP2 (49), have increased macrophage recruitment, airway ECM deposition, and reduced airway compliance (50). Therefore, therapeutics aiming to reduce cholesterol biosynthesis and/or increase cholesterol reverse transport may provide beneficial treatment for PF. However, clinical trials involving statins have not shown positive effects (51, 52). In animal experiments, BLM-induced PF is exacerbated in mice pretreated with statins, which was mediated through the NLRP3 inflammasome (51, 52). These results may be explained by the fact that statins activate SREBP2 (53). Given that SREBP2 activation in ECs is central to oxidative, inflammatory, and fibrotic pathways, agents that antagonize SREBP2 could inhibit EC-to-myofibroblast–like transition, as observed in PF. Translationally, the use of a therapeutic treatment of PF that putatively targets SREBP2 could possibly limit both the innate immune response and partial EndoMT in the lung, thereby alleviating the pathophysiological consequence due to early lung injury. In terms of clinical relevance, the potential role of SREBP2 inhibition in the treatment of patients with IPF warrants future testing. Thus far, pharmacological inhibitors of SREBP2 — i.e., betulin — are being developed; however, the physiological suppression of SREBP2 is limited by hepatic toxicity (54). Nevertheless, the current study identified a potential therapeutic target of PF, which deserves further study.

## Methods

Supplemental Methods, including Supplemental Tables 1–3, are available online with this article.

### RNA-Seq library construction and data processing.

Total RNA was isolated from HUVECs infected with adenovirus SREBP2 (Ad-SREBP2) or Ad-null (empty vector). mRNA was isolated using mirVana mRNA isolation kit (Thermo Fisher Scientific). Standard Illumina protocols were used to construct the RNA libraries and sequencing. Analysis was performed using base calling and quality scoring by using Real-Time Analysis version 2 (RTA v2) on the NextSeq 500 system. Data were demultiplexed and converted to FASTQ files using Bcl2fastq conversion software v1.8.4. Sequence reads were trimmed of their adaptor sequences and masked for low complexity or low-quality sequence. Reads were mapped to the h19 genome using tophat v2.0.14 (55). Data were normalized to reads per kilobase of transcript, per million mapped reads (RPKM) using cufflinks v2.2.1 to assemble transcripts and estimate mRNA abundance (Supplemental Figure 4) (56, 57). Data are available at NCBI’s Gene Expression Omnibus database (GSE121782).

### ATAC-Seq library construction and data processing.

Nuclei were isolated from ~5000 cells in lysis buffer (10 mM Tris-HCl, 10 mM NaCl, 3 mM MgCl_2_, and 0.1% NP-40). Nuclear pellets were resuspended in transposition buffer (Nextera DNA Library Preparation kit, Illumina) and incubated at 37°C for 30 minutes. DNA was then purified using the Qiagen MinElute PCR purification kit. Libraries were sequenced using the Illumina HiSeq 2000. ATAC-Seq data analysis followed the official pipeline of ATAqC specification of the Encyclopedia of DNA Elements (ENCODE) consortium (58). Adapter sequences were trimmed from the raw reads by using cutadapt (59). Reads were aligned to the Human GRCh38 genome using bowtie2 (60). After read alignment, SAMtools (61), Picard’s MarkDuplicates (62), and bedtools (63) were used to remove multimapped reads (mapping quality [MAPQ] < 30), remove PCR duplicates reads, and convert alignment BAM format to tagAlign format, respectively. The retained alignment results were used to call peaks by MACS2 (64) with the FDR threshold of 0.01. Results of peak signal were imported into WashU Epigenome Browser for further visualization and analysis (Supplemental Figure 5). Data are available at NCBI’s Gene Expression Omnibus database (GSE121781).

### EC culture, siRNA transfection, and adenovirus infection.

HUVECs (Lonza) were cultured at 37°C with 95% humidified air and 5% CO_2_ in M199 (Thermo Fisher Scientific) supplemented with 15% FBS (Thermo Fisher Scientific), 1 ng/mL recombinant human fibroblast growth factor (F&D Systems), 90 μg/mL heparin (Thermo Fisher Scientific), 20 mM HEPES (pH 7.4; Thermo Fisher Scientific), and 100 U/mL penicillin-streptomycin (Thermo Fisher Scientific). Lung microvascular ECs (Lonza) were cultured at 37°C with 95% humidified air and 5% CO_2_ in EGM2-MV bullet kit (Lonza). Human lung fibroblasts (Lonza) were cultured at 37°C with 95% humidified air and 5% CO_2_ in FGM2 bullet kit (Lonza). For siRNA knockdown, HUVECs and lung microvascular ECs were transfected with SREBP2 or control siRNA (25 nM) using Lipofectamine RNAiMax (Invitrogen) for up to 72 hours. SREBP2 overexpression was performed using adenovirus compared with empty vector (Ad-null) infection for 72 hours unless otherwise stated.

### Mouse lung EC isolation.

Harvested mouse lungs were immediately minced and incubated with Type 1 Collagenase (Thermo Fisher Scientific) for 45 minutes. The solution was then triturated using 12 cm cannulas, filtered at 70 μm, and centrifuged at 300*g* for 8 minutes at 4°C. Cell pellets were then resuspended in fresh M199 buffer and selected for using DynaBeads (Invitrogen) conjugated to CD31 (Santa Cruz Biotechnology Inc., clone JC70) and CD144 (BD Biosciences, clone 55-7H1) for 4 hours. Beads with bound lung ECs were then plated on collagen-coated tissue culture dishes and grown for at least 3 days prior to further experimentation.

### Mouse lines and BLM administration.

All mice were kept in a temperature- and humidity-controlled environment with a 12-hour light/dark cycle and fed ad libitum; male mice aged between 2 and 4 months were used for each study. EC-SREBP2(N)-Tg mice were generated as previously described (20). Tamoxifen inducible VE-Cad–CreERT2 mice were a gift from Kristy Red-Horse (Stanford University, Stanford, California, USA). tdTomato mice (B6.Cg-Gt[ROSA]26Sor^tm9[CAG-tdTomato]/Hze^/J) were purchased from The Jackson Laboratory (stock no. 007909). To generate EC-specific tdTomato mice (EC-tdTomato) for lineage tracing, the tamoxifen-inducible VE-Cad–CreERT2 mice were crossed with tdTomato mice. At 2 months of age, EC-tdTomato mice were i.p. injected with tamoxifen (MilliporeSigma; 75 mg/kg body weight) for 5 consecutive days and then had a 7-day waiting period prior to PF treatment. To induce PF, mice were administered a total of 5 i.p. injections of BLM (McKesson General; 8 U/kg body weight) over the course of 2 weeks (days 0, 4, 7, 10, and 14). Mice were then harvested after an additional 14 days.

### Angiography.

Immediately following euthanasia, the superior vena cava, anterior vena cava, and aorta are sutured closed. Used as the contrasting agent, Microfil (Flow Tech), was directly injected into the right ventricle to flow up the pulmonary artery and was allowed to cure overnight at room temperature. Lungs were then harvested and dehydrated in increasing percentages of absolute ethanol. Finally, lungs were immersed in methyl salicylate for 24 hours, followed by standard imaging procedures.

### Human lung sample acquisition.

Human deidentified IPF lung samples were obtained with informed consent from explants of patients with IPF undergoing lung transplantation at the UCSD. Samples from alveolar tissue at the interface between fibrosis and more normal-appearing lung were excised and processed as formalin-fixed, paraffin-embedded blocks for histopathology. Nondiseased human lungs deemed to be unsuitable for lung transplantation were obtained from beating-heart (or warm autopsy) donors through Lifesharing.

### Statistics.

All data are expressed as mean ± SEM. Two tailed Student’s *t* test was used for comparisons between 2 groups. One-way ANOVA with Bonferroni correction was used for parametric comparisons between multiple groups. Two-way ANOVA with the Kruskal-Wallis test was used for comparing nonparametric data from multiple groups. A *P* value less than 0.05 was considered to be statistically significant for all analyses.

### Study approval.

All mice and animal protocols were approved for use from the UCSD IACUC. For IPF samples, written informed consent was obtained from all subjects in accordance with the UCSD IRB. For nondiseased lungs, the UCSD IRB deemed these approaches exempt from IRB oversight, as all subjects were considered deceased.

## Author contributions

MM designed overall study, conducted experiments, acquired data, analyzed data, and wrote the manuscript. JZ, YM, MH, and JK conducted experiments and assisted in manuscript editing. HYH and CHC analyzed genome-wide data. TSH, HCH, SHS, SSW, RLH, and LW conducted experiments. SSW provided reagents and advice. RB and AM provided expertise and assisted in manuscript editing. HDH oversaw genome-wide data sets. ZBC assisted with study design and provided reagents and advice. JYJS, JSH, and MR oversaw study design, provided expertise, and assisted with manuscript editing.

## Supplementary Material

Supplemental data

## Version 1 11/22/2021

Electronic publication
