# Supplementary material for: Role of endothelial cells in pulmonary fibrosis via SREBP2 activation
Source: JCI Insight. 2021 Nov 22;6(22):e125635. doi: 10.1172/jci.insight.125635 (PMC8663776; doi:10.1172/jci.insight.125635)
Supplement: Supplemental data [file jciinsight-6-125635-s184.pdf]

Online Data Supplement

Role of Endothelial Cells in Pulmonary Fibrosis via SREBP2 Activation

Marcy Martin, Jiao Zhang, Yifei Miao, Ming He, Jian Kang, Hsi-Yuan Huang, Chih-Hung Chou, Tse-Shun Huang, Hsiao-Chin Hong, Shu-Han Su, Simon S. Wong, Rebecca L. Harper, Lingli Wang, Rakesh Bhattacharjee, Hsien-Da Huang, Zhen Chen, Atul Malhotra, Marlene Rabinovitch, James S. Hagood, and John Y-J. Shyy

Expanded Methods

Tables 1-3

Supplemental Figures 1-5

References

Expanded Methods

RNA-sequencing library construction and data processing

Total RNA was isolated from human umbilical vein endothelial cells (HUVECs) infected with Ad-SREBP2 or Ad-null (empty vector). mRNA was isolated using mirVana mRNA isolation kit (Thermo Fisher Scientific). Standard Illumina protocols were used to construct the RNA libraries and sequencing. Analysis was performed using base calling and quality scoring by using Real-Time Analysis version 2 (RTA v2) on the NextSeq 500 system. Data were demultiplexed and converted to FASTQ files using Bcl2fastq conversion software v1.8.4. Sequence reads were trimmed of their adaptor sequences and masked for low complexity or low-quality sequence. Reads were mapped to the h19 genome using tophat v2.0.14 (1). Data was normalized to RPKM using cufflinks v2.2.1 to assemble transcripts and estimate mRNA abundance (Supplemental Figure 3) (2, 3). Dataset is available at GSE121782.

ATAC-sequencing library construction and data processing

Nuclei were isolated from ~5000 cells in lysis buffer (10 mM Tris-HCl, 10 mM NaCl, 3 mM MgCl2, and 0.1% NP-40). Nuclear pellets were resuspended in transposition buffer (Nextera DNA Library Preparation kit, Illumina) and incubated at 37°C for 30 min. DNA were then purified using Qiagen MinElute PCR purification kit. Libraries were sequenced using the Illumina HiSeq 2000. ATAC-seq data analysis followed the official pipeline of ATAqC (4) specification of the Encyclopedia of DNA Elements (ENCODE) consortium. Adapter sequences were trimmed from the raw reads by using cutadapt (5). Reads were aligned to the Human GRCh38 genome using bowtie2 (6). After read alignment, SAMtools (7), Picard's MarkDuplicates (8), and bedtools (9) were used to remove multi-mapped reads ($MAPQ < 30$), remove PCR duplicates reads, and convert alignment BAM format to tagAlign format, respectively. The retained alignment results were used to call peaks by

MACS2 (10) with the false discovery rate (FDR) threshold of 0.01. Results of peak signal were imported into WashU Epigenome Browser for further visualization and analysis (Supplemental Figure 4). Dataset is available at GSE121781.

Western blot and qPCR analyses

Total protein was isolated using NP-40 and resolved by SDS-PAGE. Gels were transferred to polyvinylidene fluoride (PVDF) membranes and immunoblotted with antibodies against SREBP2 (Abcam, ab30682), snai1 (Cell Signaling, clone L70G2), αSMA (Abcam, ab124964), vimentin (Cell Signaling, clone D21H3), and β-actin (Santa Cruz Biotechnology, clone C4). Immunoblotted bands were visualized using enhanced chemiluminescence (Milipore). For qPCR, total RNA was isolated from cells or tissue using Trizol (Life Technologies). The extracted RNA was reverse transcribed using 5X PrimeScript reverse transcriptase (Takara), and normalized to β-actin as the internal control. Relative mRNA expression was determined using iQ™ SYBR Green PCR supermix (Bio-Rad) in the Bio-Rad Real-time detection system. Data was normalized using $2^{-\Delta CT}$ $_{[-(\text{CT of target gene-CT of reference gene})]}$, followed by the $2^{-\Delta CT}$ of each experimental group divided by that of the averaged control groups. Primers are listed in Table 1 below.

Table 1 primers used for qPCR assays

β-Actin	Homo sapiens	Forward: CACCAACTGGGACGACAT Reverse: ACAGCCTGGATAGCAACG
SREBP2	Homo sapiens	Forward: CCCTGGGAGACATCGACGA Reverse: CGTTGCACTGAAGGGTCCA
Snai1	Homo sapiens	Forward: CCCCAATCGGAAGCCTAA Reverse: CCTTTCCCACTGTCCTCAT

αSMA	Homo sapiens	Forward: CAGGGCTGTTTTCCCATCCAT<br>Reverse: GCCATGTTCTATCGGGTACTTC
N-Cad	Homo sapiens	Forward: AGCCAACCTTAACTGAGGAGT<br>Reverse: GGCAAGTTGATTGGAGGGATG
Vimentin	Homo sapiens	Forward: GCCCTAGACGAACTGGGTC<br>Reverse: GGCTGCAACTGCCTAATGAG
VE-Cad	Homo sapiens	Forward: CACCTTCTGCGAGGATATGG<br>Reverse: AGGAAGATGAGCAGGGTGAT
KDR	Homo sapiens	Forward: TCAGAACCCATCAGGAGTGAATACG<br>Reverse: TGCTTGGAGGTGGCTACAATCG
KLF2	Homo sapiens	Forward: AGAGTTCCCATCTCAAGG<br>Reverse: AATGCCTCTTCATGTGTAAG
Snai1	Mus musculus	Forward: GTCGTCCTTCTCGTCCACC<br>Reverse: GGCCTGGCACTGGTATCTC
αSMA	Mus musculus	Forward: ATCATGCGTCTGGACTTGG<br>Reverse: AATAGCCACGCTCAGTCAGG
N-Cad	Mus musculus	Forward: GTGGAGGCTTCTGGTGAAAT<br>Reverse: CTGCTGGCTCGCTGCTT
Vimentin	Mus musculus	Forward: CGGAAAGTGGAATCCTTGCA<br>Reverse: CACATCGATCTGGACATGCTGT
VE-Cad	Mus musculus	Forward: CCGGCGCCAAAAGAGAGA<br>Reverse: CTGGTTTTCCTTCAGCTGGAAGTGGT
Col1A1	Mus musculus	Forward: GAGAGGTGAACAAGGTCCCG<br>Reverse:

		AAACCTCTCTCGCCTCTTGC
FN	Mus musculus	Forward: GCATCAGCCCGGATGTTAGA Reverse: GTTGGTGATGAAGGGGGTCT

Tissue collection, histological and immunofluorescence analyses

The left lung was fixed in 4% paraformaldehyde (PFA), while the right lung was snap frozen in liquid nitrogen. After dehydration and OCT embedding, the blocks were sectioned at 7 μm thickness. Sections were H&E (Sigma) or Masson's trichrome (Thermo Fisher Scientific) stained according to the manufacturer's instructions. Immunofluorescence staining was performed with primary antibodies against anti-CD31 (Santa Cruz Biotechnology, clone JC70), VE-Cad (Cell signaling, clone D87F2), αSMA (Abcam, ab124964), or SREBP2 (Abcam, ab30682). After overnight incubation, slides were washed and incubated with Alexa Fluor 488-conjugatedgoat anti-mouse IgG (Life Technologies) or Alexa Fluor 568-conjugated rabbit anti-mouse IgG (Life Technologies). ImageJ was used for quantitative analysis of fluorescence intensity. For in vivo immunofluorescence studies, the number or αSMA, vimentin, or SREBP2 positive cells were compared to the total number of VE-Cad or tdTomato positive cells in the intima of the vessel wall. Percentage of positive cells were then graphed for quantitation.

MSP-qPCR

Genomic DNA was isolated using QIAamp DNA Mini Kit (Qiagen) from indicated cells, and then bisulfite converted using EpiTect Bisulfite kit (Qiagen). Following bisulfite conversion, DNA methylation status was quantified by qPCR with primers (listed below in Table 2) that specifically recognize the methylated cytosine. UBB was used as an internal control.

Table 2 primers used for MSP assays

UBB	Homo sapiens	Forward: ATAGTGGGTTTTGTTGATTTGA Reverse: CCTTTCTCACACTAAAATTCCA
VE-Cad	Homo sapiens	Forward: GGTACGTAGATTATGAGGATAGGC Reverse: AAAATAAACAAACAACAACTCAACG
KDR	Homo sapiens	Forward: AGAGTTTTTGAGAGGTTTCGATATC Reverse: CCTTACGCTAACAACCCGAC
KLF2	Homo sapiens	Forward: GTACGTTTGTAATTTTCGTTATTCG Reverse: ATAAAACAAAATCTCGCTATATCGC

Promoter binding site prediction

Sterol regulatory elements (SREs) that SREBP2 putatively binds was predicted on snai1, aSMA, N-Cad, and vimentin promoter regions (-1000 to +500 from the transcriptional start sites [TSS]) using the weight matrix-based program MATCH (11). CpG islands were predicted using WashU EPI Genome Browser.

Chromatin immunoprecipitation (ChIP)

ChIP assays were performed according to established protocol (12) using anti-SREBP2 (Abcam, ab30682), anti-H3K27ac (Abcam, ab4729), and the isotype control anti-rabbit IgG (Santa Cruz). Briefly, ECs were cross-linked with 0.75% formaldehyde at room temp for 10 min and quenched with 125 mM glycine. Cells were lysed in RIPA buffer and sonicated to fragment the DNA to a size of 500-1000 bp. Cell lysates were then incubated with Dynabeads (Invitrogen) conjugated to the indicated antibodies. Immunoprecipitated DNA fragments were reverse cross-linked and DNA binding was quantified as percentage of input using qPCR (see list of primers below in Table 3).

Table 3 primers used for ChIP assays

Snai1-ChIP	Homo sapiens	Forward: GCTACGCGTGGATCGAGGATCTTCACGGG Reverse: GCTAAGCTTAGAAGAACCACTCGCTAGGC
αSMA-ChIP	Homo sapiens	Forward: GCTACGCGTTCTTTCGAGCACCTTCCGAG Reverse: GCTAAGCTTCGCCTCGTTCAGCGACT
N-Cad-ChIP	Homo sapiens	Forward: GCTACGCGTCCGGAGAACAGTCTCCAACT Reverse: GCTAAGCTTGCGAGCTGATGACAAATAGCG
Vimentin-ChIP	Homo sapiens	Forward: GCTACGCGTAACTTAGGGGCGCTCTTGTC Reverse: GCTAAGCTTAGAGCGCCTGAGATTGGAAC

Luciferase Assay

The 5'-upstream regions of human *snai1, αSMA, N-Cad, and vimentin* genes were PCR-amplified using KOD hot start high-fidelity DNA polymerase (Milipore) with human genomic DNA as the template. The DNA fragment spanning from (-937 to -505 bp for *SNAI1*, 53 to 124 bp for *ACTA2*, 272 to 837 bp for *CDH2*, and 82 to 914 bp for *VIM*) were subcloned into the pGL3-basic plasmid containing the firefly luciferase reporter gene (Promega), and verified by sanger sequencing. Deletions The reporter plasmids were co-transfected with Renilla into ECs, using lipofectamine 2000 (Life Technologies) for 16 hr. The transfected ECs were then infected with Ad-SREBP2 or Ad-null for an additional 48 hr. Luciferase activities were measured and normalized to Renilla activity using the Dual-Glo Luciferase Reporter Assay kit (Promega).

DNMT1 Activity assay

DNMT1 activity was measured using a DNMT1 Assay kit (Abcam) following the manufacturer's instructions. Briefly, nuclear extracts of BLM treated HUVECs were isolated using a Nuclear Extraction kit (Abcam). Nuclear extracts were then incubated with capture reagents and antibodies

specific to active DNMT1 using ELISA. Colorimetric intensity was used to measure DNMT1 activity.

Lung EC isolation

Harvested lungs were immediately minced and incubated with Type 1 Collagenase (Thermo Fisher Scientific) for 45 min. The solution was then triturated using 12 cm cannulas, filtered at 70 µm, and spun down at 8000 rpm for 8 min at 4°C. Cell pellets were then resuspended in fresh M199 buffer and selected for using DynaBeads (Invitrogen) conjugated to CD31 (Santa Cruz Biotechnology, clone JC70) and CD144 (BD Biosciences, clone 55-7H1) for 4 hr. Beads with bound lung ECs were then plated on collagen coated tissue culture dishes and grown for at least three days prior to further experimentation.

Supplemental Figure 1

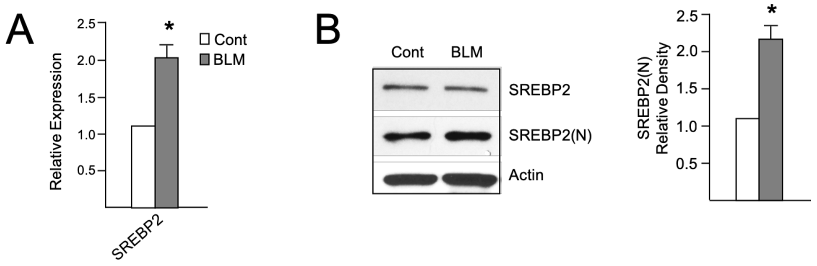

Supplemental Figure 1. Bleomycin (BLM) activates SREBP2(N) in human lung microvascular endothelial cells (HLMVECs). HLMVECs were treated with BLM (1 mU) for 24 hrs. **(A)** The mRNA expression levels of SREBP2 was measured using qPCR. **(B)** SREBP2(N) cleavage was determined by Western blot. Data in (A, B) were analyzed by two tailed Student's *t* test, error bars represent mean±SEM from three independent experiments (n=3). *P <0.05 between the indicated groups.

Supplemental Figure 2

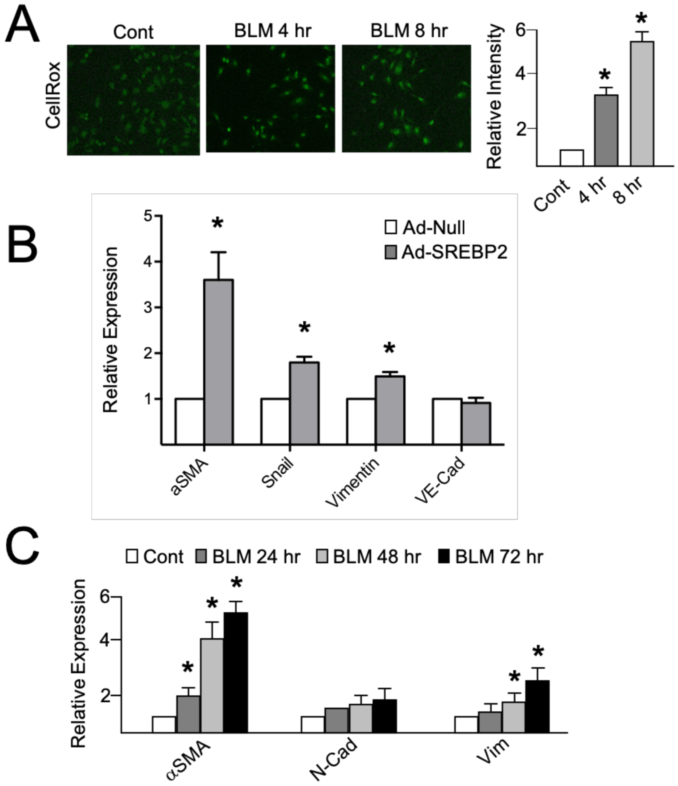

Supplemental Figure 2. Bleomycin (BLM) induces intracellular ROS and mesenchymal gene expression. (A) HUVECs were treated with BLM (1 mU) at increasing time intervals. Representative images of CellRox stain indicating intracellular ROS (green), quantification is graphed on the right. **(B)** Lung microvascular ECs were infected with Ad-SREBP2 or empty vector (Ad-Null) for 72 hrs. mRNA expression was measured by qPCR. **(C)** HUVECs treated with BLM (1 mU) at increasing time intervals, levels of mRNA were quantified using qPCR. Data in (A, B) were analyzed by two tailed Student's *t* test, error bars represent mean±SEM from three independent experiments (*n*=3). Data in (C) was analyzed by one-way ANOVA with Bonferroni post-hoc, error bars represent mean±SEM from three independent experiments (*n*=3). *P <0.05 between the indicated groups.

Supplemental Figure 3

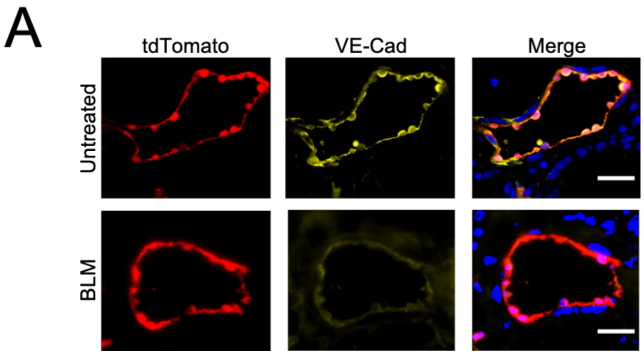

Supplemental Figure 3. Endothelial cell (EC) specificity of tamoxifen inducible tdTomato lineage tracing mice. tdTomato mice (B6.Cg-Gt(ROSA)26Sor$^{tm9(CAG-tdTomato)/Hze}$/J) were crossed with a tamoxifen inducible VE-Cad cre mice (VE-cadherin-CreERT2) to produce EC specific expression of tdTomato (EC-tdTomato mice). At 2 months of age, EC-tdTomato mice were i.p. injected with tamoxifen (75 mg/kg body weight) for 5 consecutive days. Mice were either sacrificed 7 days post injection to determine tdTomato localization compared to VE-cad immunostaining (untreated), or were administered BLM (8 units/injection) on days 0, 4, 7, 10, and 14 through i.p. injection. On 28-day post BLM administration, lungs were harvested. **(A)** Representative image of tdTomato (red) colocalization with VE-Cad (yellow) staining (n=3 mice per group). Nuclei are counterstained with DAPI (blue). Scale bar is 20 microns.

Supplemental Figure 4

A

Sample ID	RawData	QC	MapToGenome	QC/Raw	Map/QC	Map/Raw
Ad-Null-1	23204750	22481332	21126949	96.9%	94.0%	91.0%
Ad-Null-2	18619401	17999733	16924378	96.7%	94.0%	90.9%
Ad-SREBP2-1	20134085	19581421	18559018	97.3%	94.8%	92.2%
Ad-SREBP2-2	22149266	21501495	20390922	97.1%	94.8%	92.1%

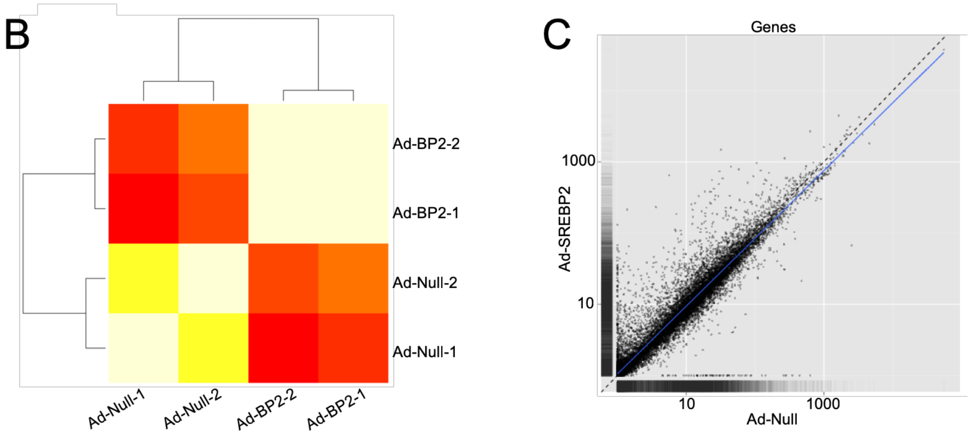

Supplemental Figure 4. Overall RNA-seq statistics and mapping for SREBP2 overexpression. HUVECs were infected with Ad-Null or Ad-SREBP2 for 72 hrs. (**A**) Mapping statistics of RNA-seq data demonstrating that more than 90% of reads were mapped to the genome. **(B)** Data distribution between the two sets of biological repeats. **(C)** Scatter plot identifying global transcript changes between Ad-Null and Ad-SREBP2 biological duplicates.

Supplemental Figure 5

A

	SREBP2_1	SREBP2_2	SREBP2_3	Null_1	Null_2	Null_3
Read count from sequencer	52,490,758	53,774,414	58,632,676	53,440,364	55,553,204	45,239,858
Read count successfully aligned	49,949,071	50,971,751	53,971,989	53,063,482	55,127,056	44,863,316
Read count after filtering for mapping quality	32,338,576	33,550,433	34,432,371	36,034,293	32,720,929	29,108,450
Read count after removing duplicate reads	27,318,110	28,802,352	27,981,578	31,045,927	25,906,250	24,687,956
Read count after removing mitochondrial reads	21,790,328	23,483,500	20,865,180	25,472,180	18,518,452	19,762,704

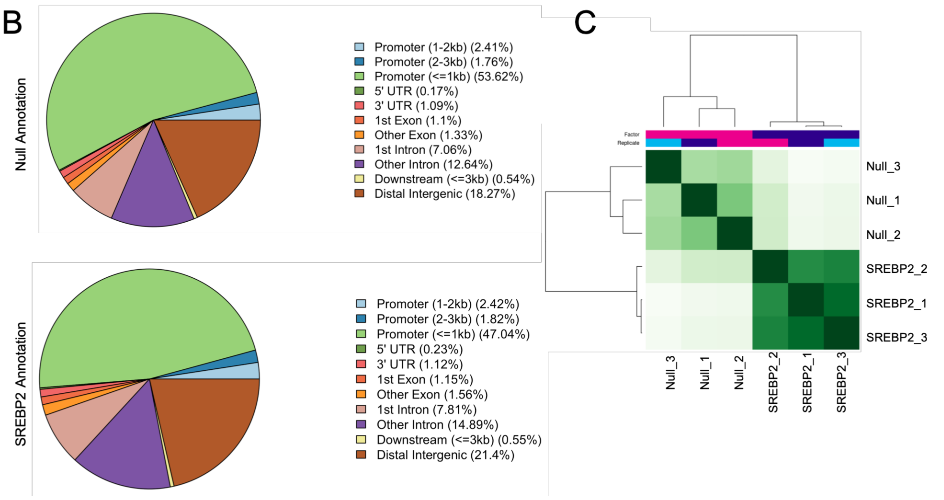

Supplemental Figure 5. Overall ATAC-seq statistics and mapping for SREBP2 overexpression. HUVECs were infected with Ad-Null or Ad-SREBP2 for 72 hrs. (**A**) Mapping statistics data. **(B)** A genome-wide ATAC-seq annotation illustrating the areas of chromosome decondensation **(C)** Principal component clustering indicating similarity between biological triplicates.

References

1. Kim D, Pertea G, Trapnell C, Pimentel H, Kelley R, Salzberg SL. TopHat2: accurate alignment of transcriptomes in the presence of insertions, deletions and gene fusions. *Genome biology* 2013; 14: R36.
2. Roberts A, Trapnell C, Donaghey J, Rinn JL, Pachter L. Improving RNA-Seq expression estimates by correcting for fragment bias. *Genome biology* 2011; 12: R22.
3. Trapnell C, Williams BA, Pertea G, Mortazavi A, Kwan G, van Baren MJ, Salzberg SL, Wold BJ, Pachter L. Transcript assembly and quantification by RNA-Seq reveals unannotated transcripts and isoform switching during cell differentiation. *Nature biotechnology* 2010; 28: 511-515.
4. Kundaje Lab, ATAqC: ATAC-seq processing pipeline. https://github.com/kundajelab/atac_dnase_pipelines. 2016.
5. Martin M. Cutadapt removes adapter sequences from high-throughput sequencing reads. *2011* 2011; 17.
6. Langmead B, Salzberg SL. Fast gapped-read alignment with Bowtie 2. *Nat Methods* 2012; 9: 357-359.
7. Li H, Handsaker B, Wysoker A, Fennell T, Ruan J, Homer N, Marth G, Abecasis G, Durbin R, Genome Project Data Processing S. The Sequence Alignment/Map format and SAMtools. *Bioinformatics* 2009; 25: 2078-2079.
8. Institute B. Picard Tools. https://broadinstitute.github.io/picard/. 2016.
9. Quinlan AR, Hall IM. BEDTools: a flexible suite of utilities for comparing genomic features. *Bioinformatics* 2010; 26: 841-842.

10. Zhang Y, Liu T, Meyer CA, Eeckhoute J, Johnson DS, Bernstein BE, Nusbaum C, Myers RM, Brown M, Li W, Liu XS. Model-based analysis of ChIP-Seq (MACS). *Genome Biol* 2008; 9: R137.

11. Kel AE, Gossling E, Reuter I, Cheremushkin E, Kel-Margoulis OV, Wingender E. MATCH: A tool for searching transcription factor binding sites in DNA sequences. *Nucleic acids research* 2003; 31: 3576-3579.

12. Heintzman ND, Hon GC, Hawkins RD, Kheradpour P, Stark A, Harp LF, Ye Z, Lee LK, Stuart RK, Ching CW, Ching KA, Antosiewicz-Bourget JE, Liu H, Zhang X, Green RD, Lobanenkov VV, Stewart R, Thomson JA, Crawford GE, Kellis M, Ren B. Histone modifications at human enhancers reflect global cell-type-specific gene expression. *Nature* 2009; 459: 108-112.
